# Front-of-Package Food Labels and Perceived Weight Stigmatization: A Randomized Clinical Trial

**DOI:** 10.1001/jamanetworkopen.2025.16821

**Published:** 2025-06-20

**Authors:** Aline D’Angelo Campos, Anna H. Grummon, Shu Wen Ng, Rebecca M. Puhl, Shelley D. Golden, Marissa G. Hall

**Affiliations:** 1 Department of Health Behavior, Gillings School of Global Public Health, University of North Carolina at Chapel Hill; 2 Carolina Population Center, University of North Carolina at Chapel Hill; 3 Department of Pediatrics, Stanford University School of Medicine, Palo Alto, California; 4 Department of Health Policy, Stanford University School of Medicine, Stanford, California; 5 Department of Nutrition, Gillings School of Global Public Health, University of North Carolina at Chapel Hill; 6 Department of Human Development & Family Sciences, University of Connecticut, Storrs; 7 Rudd Center for Food Policy and Health, University of Connecticut, Hartford; 8 Lineberger Comprehensive Cancer Center, University of North Carolina at Chapel Hill

## Abstract

**Importance:**

Front-of-package food labels (FOPLs) about nutrient content and health effects are a promising policy to improve diet quality but may also contribute to harmful weight stigma.

**Objective:**

To assess whether different types of FOPLs impact perceived weight stigmatization, whether weight-neutral label content mitigates stigmatization, and possible trade-offs between perceived stigmatization and effectiveness.

**Design, Setting, and Participants:**

This randomized clinical trial with a between- and within-participant design used a single-exposure online survey conducted from January 18 to 26, 2024. Participants were US adults aged 21 years or older recruited using an online crowdsourcing research platform.

**Interventions:**

Participants were randomly assigned 1:1:1:1 to 1 of 4 types of FOPLs applied to sugary beverages: control labels, nutrient warnings (ie, indicating beverages high in calories or added sugar), text-only health warnings, or graphic health warnings (ie, indicating beverages were linked to obesity, diabetes, and tooth decay). In random order, participants viewed 2 versions of their assigned label type differing on whether the content referenced calories and obesity (ie, standard version) or not (ie, weight-neutral version).

**Main Outcome and Measures:**

The primary as-treated outcome was perceived weight stigmatization. Secondary outcomes were perceived message effectiveness, attributional judgments of responsibility for weight, and explicit weight bias.

**Results:**

A total of 2522 participants completed the experiment (1262 [50%] women; mean [SD] age, 44.3 [15.2] years). Among standard labels, graphic warnings (mean differential effect [MDE], 0.81; 95% CI, 0.71-0.92) and text-only warnings (MDE, 0.41; 95% CI, 0.30-0.51) were perceived as more stigmatizing than control labels, while nutrient warnings were not (MDE, 0.003 [95% CI, −0.10 to 0.11]; *P* = .96). Weight-neutral labels were perceived as less stigmatizing than their respective standard versions (range: MDE, −0.66 [95% CI, −0.72 to −0.60] for graphic health warnings to −0.08 [95% CI, −0.14 to −0.02] for nutrient warnings). Weight-neutral versions were perceived as less effective than standard versions for nutrient warnings (MDE, −0.11; 95% CI, −0.16 to −0.06) and graphic health warnings (MDE, −0.05; 95% CI, −0.10 to −0.001) but not for text-only warnings. Graphic and text-only warnings did not affect explicit weight bias, while nutrient labels led to a small bias reduction (MDE, −0.08; 95% CI, −0.16 to −0.002).

**Conclusions and Relevance:**

In this randomized clinical trial of FOPLs, nutrient warnings performed best compared with other FOPL types at simultaneously maximizing perceived effectiveness and minimizing perceived stigmatization. Labels perceived as more stigmatizing were not consistently perceived as more effective. Removing references to obesity from health warnings reduced stigmatization without meaningfully reducing perceived effectiveness.

**Trial Registration:**

ClinicalTrials.gov Identifier: NCT06179043

## Key Points

**Question:**

Do different front-of-package food labels (FOPLs) impact weight stigmatization?

**Findings:**

In this randomized clinical trial of 2522 adults, participants did not rate FOPL nutrient warnings on sugar-sweetened beverages as significantly more stigmatizing than control labels. Participants rated text-only and graphic health warnings as significantly more stigmatizing than control labels when obesity was referenced; when obesity was not referenced, participants rated only graphic health warning labels as more stigmatizing than control labels.

**Meaning:**

Nutrient and text-only health warnings that did not mention obesity were not rated as more stigmatizing than control labels.

## Teaser

This randomized clinical trial investigates whether front-of-package food labels with nutrient or health warning affect perceived weight stigmatization and effectiveness at discouraging purchase.

## Introduction

Weight stigma, also referred to as weight bias, refers to negative attitudes, stereotypical beliefs, and societal devaluation of people with high body weight.^1^ Weight stigma is widespread: among US youths, weight is the most common reason for bullying,^2^ while adults face weight-based discrimination (ie, behavioral manifestation of stigma) in professional,^3,4^ health care,^3,5^ and interpersonal settings.^3,6^ Stress experienced in response to weight stigma can contribute to maladaptive coping mechanisms, including unhealthy eating,^7,8,9,10^ exercise avoidance,^10,11^ health care delays,^12,13,14^ higher cortisol reactivity,^15,16,17^ and systemic inflammation.^18^ Experiencing weight stigma is a risk factor for mental and physical health issues, including weight gain,^19,20,21^ depression,^22,23,24^ anxiety,^23,24^ eating disorders,^25,26,27^ and suicidality.^28,29,30^

Given the harms caused by weight stigma, public health interventions should avoid reinforcing it. Avoiding stigmatization is particularly important for interventions seeking to influence individual dietary behavior, which may inadvertently reinforce perceptions that individuals are responsible for their weight,^31^ thereby contributing to stigma.^32,33,34^ One intervention seeking to influence dietary behavior gaining traction worldwide is interpretive front-of-package labeling (FOPLs) of foods and beverages. Interpretive FOPLs go beyond factual nutritional information and help consumers interpret products’ nutritional quality. Studies show that well-designed interpretive FOPLs can positively influence consumer choices^35,36,37,38^ and prompt manufacturers to reformulate products.^39,40^ The World Health Organization^41^ recommends FOPL policies, and 15 countries currently mandate some type of interpretive FOPL.^42^ Among these, the most common type are nutrient warnings, which warn consumers when products are high in nutrients of concern (eg, sugar, saturated fat, and sodium). In 2025, the US Food and Drug Administration (FDA) proposed mandatory FOPLs that, despite not constituting warnings, would similarly signal foods high in nutrients of concern.^43^ Health warnings,^44^ which warn consumers that products are linked to health issues, are another type of interpretive FOPL previously proposed for sugar-sweetened beverages in 7 states and 2 cities in the US.

While evidence from policy evaluations comparing nutrient and health warnings is scarce, laboratory studies suggest that health warnings may be more effective than nutrient warnings.^35^ However, no studies to our knowledge have compared the weight stigmatization posed by these different types of FOPLs, and previous studies showed that only some types of FOPLs, including graphic health warnings and octagonal nutrient warnings, were perceived as more stigmatizing than control conditions.^45,46^ To address this gap, we examined whether different FOPLs affect perceived weight stigmatization and whether making label content weight neutral could mitigate stigmatization. We also assessed perceived effectiveness of FOPLs to examine possible trade-offs between stigmatization and effectiveness within the same sample and stimuli.

## Methods

### Participants

In this randomized clinical trial, from January 18 to 26, 2024, we recruited an online convenience sample of adults aged 21 years or older residing in the US using CloudResearch’s Connect platform.^47^ Participants received $1.25 for completing the survey. This study followed a parent study examining messages to discourage alcohol consumption, none of which referenced body weight.^48^ We included all participants from the parent study. The Stanford University institutional review board approved this study. Participants provided written informed consent. The study design, measures, hypotheses, and analytic plan were registered prior to data collection (NCT06179043). We followed the Consolidated Standards of Reporting Trials (CONSORT) reporting guideline for clinical trials. The trial protocol is given in Supplement 1.

### Procedures

**Figure 1.**
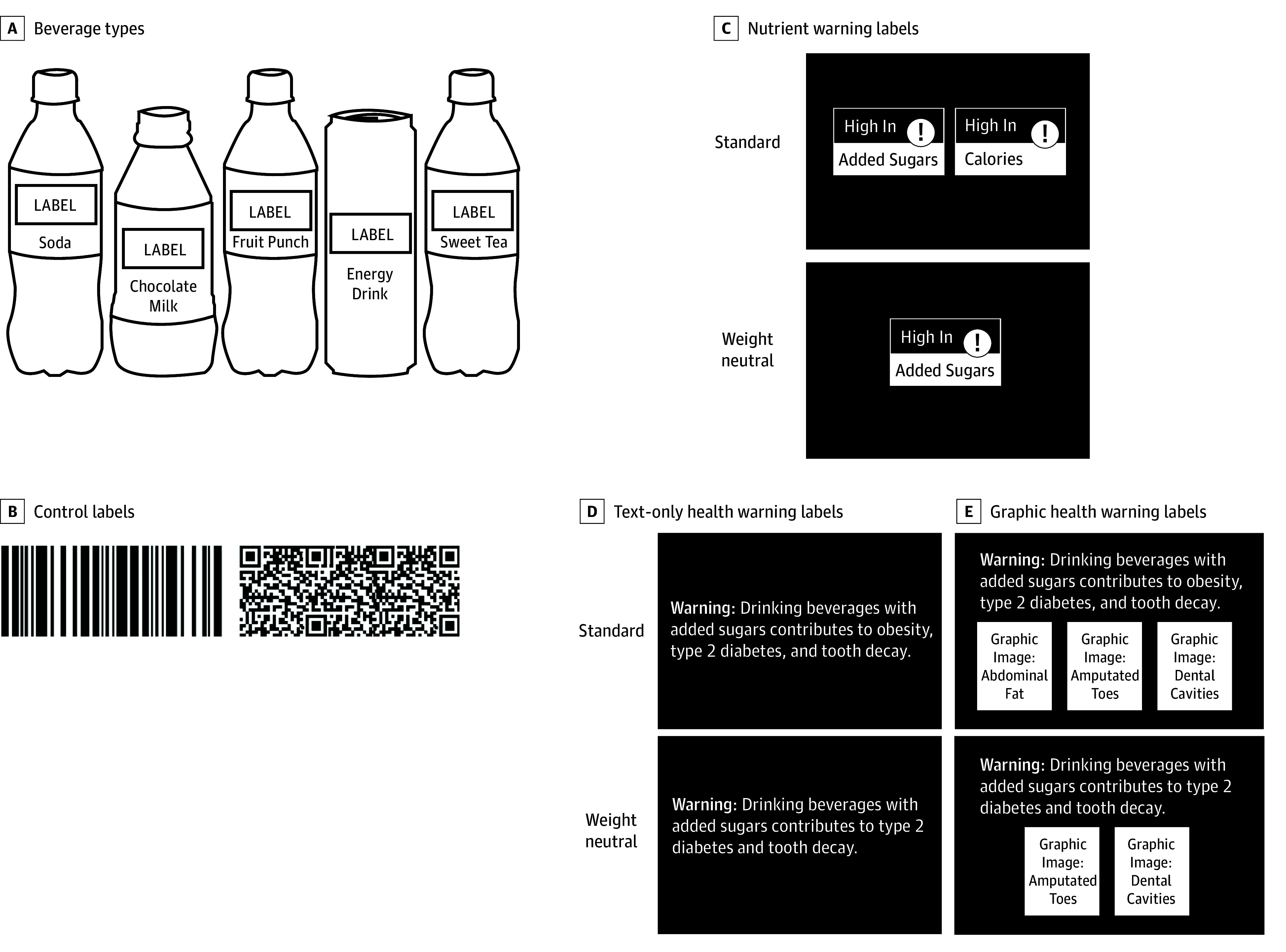
Study Stimuli and Experimental Design Stimuli were edited to comply with journal policies. A link to the original stimuli used in the study is given in the eAppendix in Supplement 2.

Participants completed an online experiment using a 3  × 2 plus control, between- and within-participant design. Participants viewed several beverages simultaneously, each displaying a randomly assigned label, to illustrate the context in which participants would encounter the labels. A callout box showed the augmented label (Figure 1).

The between-participants factor was label type. Participants were randomly assigned 1:1:1:1 to 1 of 4 label types during the survey: control, nutrient warnings, text-only health warnings, or graphic health warnings. Control labels were neutral barcodes and QR codes matching the size and shape of other labels, following prior studies.^49,50^ Nutrient warnings were based on designs under FDA consideration at the time of data collection, with an added exclamation point to enhance interpretability.^51,52^ Both types of health warnings used the language proposed by state and local governments, including the word *warning*.^44^ Graphic warnings also contained images depicting the referenced health outcomes, following a prior study.^45^

The within-participant factor was label version. Participants in the noncontrol arms saw 2 versions of their assigned label type in random order: standard and weight neutral. Weight-neutral versions omitted references to body weight or nutritional characteristics exclusively related to weight regulation. Specifically, standard nutrient warnings stated that products were “high in added sugars” and “high in calories,” while the weight-neutral version omitted the calorie statement. Standard text-only and graphic health warnings stated, “Drinking beverages with added sugars contributes to obesity, type 2 diabetes, and tooth decay,” while the weight-neutral versions omitted the word *obesity*. The standard graphic health warning displayed an image of an abdomen, while the weight-neutral version did not (Figure 1).

### Measures

In random order, participants viewed the 2 versions of their randomly assigned label type and rated their perceptions of each (eTable 1 in Supplement 2). First, participants rated perceived weight stigmatization (PWS)—that is, how much they perceived the label as stigmatizing, promoting negative stereotypes, or disrespecting people with obesity. The 3 items were adapted from a measure evaluating how generally stigmatizing (ie, without distinguishing between explicit vs internalized stigma) health messages are perceived to be toward people with obesity.^53^ Assessing message perceptions is rooted in the Elaboration Likelihood Model of persuasion, which posits that message perceptions shape attitudes.^54^ We chose items that allowed for comparisons with prior studies using the same items.^45,46^ Next, participants rated each label’s perceived message effectiveness (PME)—that is, how much the label discouraged them from buying sugar-sweetened beverages. Validation studies show that PME is predictive of behavior change in the context of label exposure.^55,56^

After rating the labels, participants reported their direct judgments and biases related to body weight (eTable 1 in Supplement 2). First, 2 items adapted from a previous study^57^ assessed participants’ attributional judgments (ie, the belief that people are responsible for their weight), which represent the main mechanism through which FOPLs are likely to affect weight stigma.^31^ Next, 7 items from the validated Fat Phobia Scale^58^ assessed participants’ explicit weight bias—that is, their conscious, self-reported endorsement of stereotypes about people with high body weight.^59^ Items reflected salient stereotypes associated with high body weight: laziness, lack of willpower and self-discipline, inactivity, and gluttony.^3^ For all measures, response scales ranged from 1 (coded as “low”) to 5 (coded as “high”). In addition, the survey collected demographic characteristics. Race and ethnicity, ascertained by self-report, were included in the analysis because previous evidence suggests that people in different racial and ethnic groups exhibit different levels of internalized weight stigma.^60,61,62^ Categories were Hispanic, Latino, or of Spanish origin; non-Hispanic American Indian or Alaska Native; non-Hispanic Asian; non-Hispanic Black or African American; non-Hispanic Middle Eastern or North African; non-Hispanic White; and other non-Hispanic race (not broken down further because doing so would likely lead to cell sizes too small for analysis).

### Statistical Analysis

Although the parent study determined the sample size, we estimated minimum detectable effect sizes using G*Power, version 3.1 (University of Dusseldorf). Assuming a 2-sided critical α of .05 and 2 repeated measures, a sample of 2500 yielded 80% power to detect minimum effects of Cohen *d* = 0.11 for the between-participants factor and Cohen *d* = 0.06 for the within-participant factor. These effects were conservative based on nutrient warnings research.^46^

Participants who did not complete the survey or completed the survey implausibly quickly (ie, less than one-third of the median completion time) were excluded per the parent study’s registration.^48^ For measures with multiple items, we verified sufficient internal reliability (α = .94 for PWS, α = .87 for explicit weight bias, and α = .85 for attributional judgments) and averaged responses across items. To examine impacts on PWS and PME, we fit linear mixed-effects models with random intercepts to account for repeated measures within participants (eTable 2 in Supplement 2). Models regressed outcomes on indicator variables representing the 2 control labels and each combination of experimental factors. After estimation, we performed Wald tests to examine interactions between experimental factors. Due to significant interactions between factors for the primary outcome (*P* < .001), we present mean differential effects (MDEs; ie, pairwise differences in mean scores between experimental conditions) between label types (pooling control labels) for standard and weight-neutral labels separately. Additionally, we present MDEs between the standard and weight-neutral versions of each label type. To examine impacts on attributional judgments and explicit weight bias, we fit linear regression models including indicator variables representing label types (eTable 2 in Supplement 2). We present MDEs between label types. For all models, we used the Bonferroni-Holm correction for comparisons that did not involve the control arm (3 tests per outcome: nutrient vs text only, nutrient vs graphic, and text only vs graphic).

Previous evidence suggests that youths and women experience weight stigma disproportionately,^2,60,63,64^ while Black and Hispanic individuals are less likely than non-Hispanic White individuals to internalize weight stigma.^60,61,62^ Additionally, people with higher vs lower body weight are conceptually more likely to have experienced weight stigma. Because these experiences may influence label perceptions, we conducted exploratory analyses to examine whether gender, race and ethnicity, perceived weight status, or age moderated the effects of labels on PWS. Due to significant interactions between label type and version in the main model, we fit separate mixed-effects moderation models for standard and weight-neutral labels. Models included indicator variables representing the 2 control labels and each combination of noncontrol labels with moderator levels. We used postestimation Wald tests to examine interactions between label type and the moderator and calculated marginal effects of labels on PWS (compared with pooled control) at each moderator level. We also conducted exploratory (post hoc) analyses examining whether attributional judgments and other theory-based drivers of weight stigma mediated the effects of FOPLs on explicit weight bias (eAppendix in Supplement 2).

We used complete case analysis, resulting in the exclusion of 1 participant from 1 model. Analyses were conducted using Stata/SE, version 17 (StataCorp LLC) with a 2-sided critical α of .05. Experimental analysis made 2 deviations from the registered analytic plan. First, although post hoc, we examined effects on attributional judgments given their strong role in weight stigma.^32,33,34,65^ Second, the registered protocol indicated that we would regress outcomes on indicators for experimental factors and their interaction. We instead regressed outcomes on indicator variables for each combination of experimental factors and the separate control conditions, as recommended when the control group does not fit the factorial design.^66^

## Results

**Figure 2.**
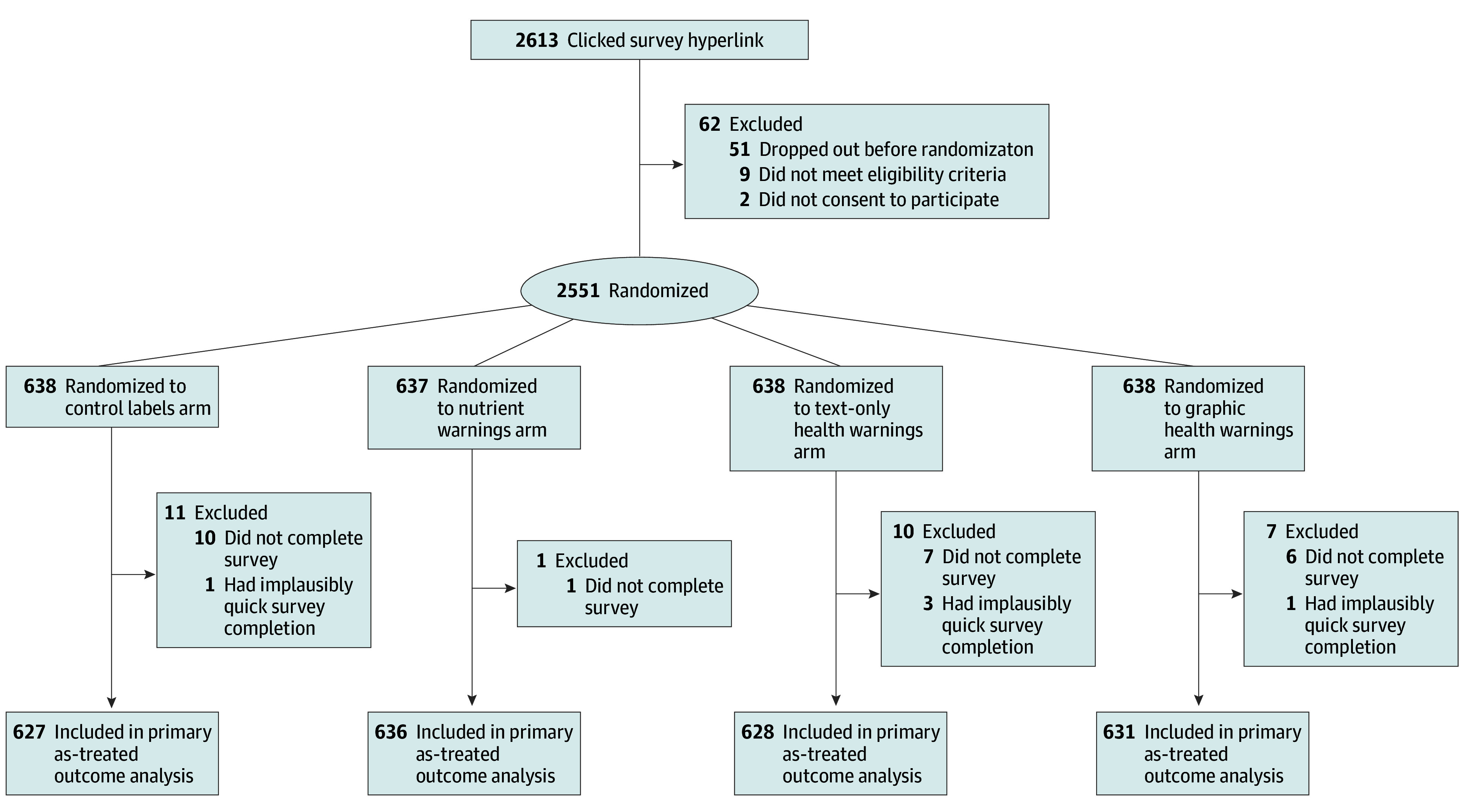
Participant Flow Diagram

A total of 2551 individuals were randomized (638 each to the control label, text-only health warning, and graphic health warning arms and 637 to the nutrient warning arm). Among randomized participants, those who did not complete the survey (n = 24) or completed the survey implausibly quickly (n = 5) were excluded. The as-treated analysis included 2522 participants (Figure 2). The mean (SD) age was 44.3 (15.2) years; of 2521 participants reporting gender, 1252 (50%) identified as men, 5 (<1%) identified as nonbinary, 1262 (50%) identified as women, and 2 (<1%) preferred to self-describe their gender. Of 2521 reporting race and ethnicity, 6 (<1%) identified as American Indian or Alaska Native; 117 (5%) as Asian; 318 (13%) as Black or African American; 178 (7%) as Hispanic, Latino, or of Spanish origin; 4 (<1%) as Middle Eastern or North African; 1890 (75%) as White; and 8 (<1%) as other race. Half (1273 of 2521 [50%]) perceived themselves as slightly or very overweight, and 1502 of 2520 (60%) had a bachelor’s degree or higher (Table). The sample was similar to the US adult population in distribution of gender and most income brackets but included higher proportions of individuals with higher educational level and White people (eTable 3 in Supplement 2).

**Table.** Sample Characteristics

Characteristic	Participants^a^				
	Full sample (N = 2522)	Control arm (n = 627)	Nutrients arm (n = 636)	Text only arm (n = 628)	Graphic arm (n = 631)
Gender (n = 2521)^b^					
Man	1252 (50)	304 (48)	319 (50)	314 (50)	315 (50)
Nonbinary	5 (<1)	0	1 (<1)	3 (<1)	1 (<1)
Woman	1262 (50)	323 (52)	315 (50)	309 (49)	315 (50)
Prefer to self-describe	2 (<1)	0	0	2 (<1)	0
Race and ethnicity (n = 2521)^b^					
American Indian or Alaska Native, non-Hispanic	6 (<1)	1 (<1)	0	3 (<1)	2 (<1)
Asian, non-Hispanic	117 (5)	35 (6)	31 (5)	28 (4)	23 (4)
Black or African American, non-Hispanic	318 (13)	75 (12)	90 (14)	75 (12)	78 (12)
Hispanic, Latino, or of Spanish origin	178 (7)	40 (6)	45 (7)	53 (8)	40 (6)
Middle Eastern or North African, non-Hispanic	4 (<1)	4 (1)	0	0	0
White, non-Hispanic	1890 (75)	472 (75)	467 (74)	463 (74)	488 (77)
Other, non-Hispanic^c^	8 (<1)	0	2 (<1)	6 (1)	0
Educational level (n = 2521)^b^					
<High school	14 (1)	3 (<1)	3 (<1)	6 (1)	2 (<1)
High school or GED	264 (10)	69 (11)	57 (9)	73 (12)	65 (10)
Some college or technical school	472 (19)	118 (19)	118 (19)	114 (18)	122 (19)
Associate’s degree	269 (11)	69 (11)	67 (11)	82 (13)	51 (8)
Bachelor’s degree	1032 (41)	242 (39)	273 (43)	243 (39)	274 (43)
Graduate or professional degree	470 (19)	126 (20)	117 (18)	110 (18)	117 (19)
Sexual orientation (n = 2520)^d^					
Straight or heterosexual	2186 (87)	557 (89)	531 (84)	540 (86)	558 (88)
Gay or lesbian	101 (4)	21 (3)	36 (6)	24 (4)	20 (3)
Bisexual	202 (8)	43 (7)	59 (9)	54 (9)	46 (7)
Prefer to self-describe	31 (1)	6 (1)	9 (1)	9 (1)	7 (1)
Perceived weight status (n = 2521)^b^					
Very underweight	14 (1)	5 (1)	3 (<1)	3 (<1)	3 (<1)
Slightly underweight	144 (6)	38 (6)	29 (5)	39 (6)	38 (6)
About the right weight	1090 (43)	276 (44)	276 (43)	253 (40)	285 (45)
Slightly overweight	989 (39)	226 (36)	250 (39)	263 (42)	250 (40)
Very overweight	284 (11)	82 (13)	77 (12)	70 (11)	55 (9)
Annual household income, $ (n = 2519)^e^					
<10 000	76 (3)	18 (3)	20 (3)	13 (2)	25 (4)
10 000-14 999	69 (3)	8 (1)	14 (2)	26 (4)	21 (3)
15 000-24 999	171 (7)	37 (6)	38 (6)	51 (8)	45 (7)
25 000-34 999	230 (9)	55 (9)	58 (9)	64 (10)	53 (8)
35 000-49 999	346 (14)	95 (15)	84 (13)	77 (12)	90 (14)
50 000-74 999	558 (22)	143 (23)	135 (21)	143 (23)	137 (22)
75 000-99 999	421 (17)	104 (17)	117 (18)	101 (16)	99 (16)
100 000-149 999	403 (16)	113 (18)	93 (15)	97 (15)	100 (16)
150 000-199 999	132 (5)	26 (4)	40 (6)	32 (5)	34 (5)
≥200 000	113 (4)	27 (4)	36 (6)	24 (4)	26 (4)
Age, mean (SD), y	44.3 (15.2)	44.7 (15.1)	43.3 (15.2)	44.6 (14.9)	44.4 (15.4)
Individuals in household, mean (SD), No. (n = 2521)^b^	2.6 (1.4)	2.6 (1.3)	2.5 (1.3)	2.6 (1.4)	2.6 (1.4)

Abbreviation: GED, General Educational Development.

^a^ Data are presented as number (percentage) of participants unless otherwise indicated.

^b^ Data were missing for 1 participant in the nutrients arm.

^c^ “Other” was not broken down further because doing so would likely lead to cell sizes too small for analysis.

^d^ Data were missing for 1 participant in the nutrients arm and 1 in the text-only arm.

^e^ Data were missing for 1 participant each in the control, nutrients, and graphic arms.

### PWS

**Figure 3.**
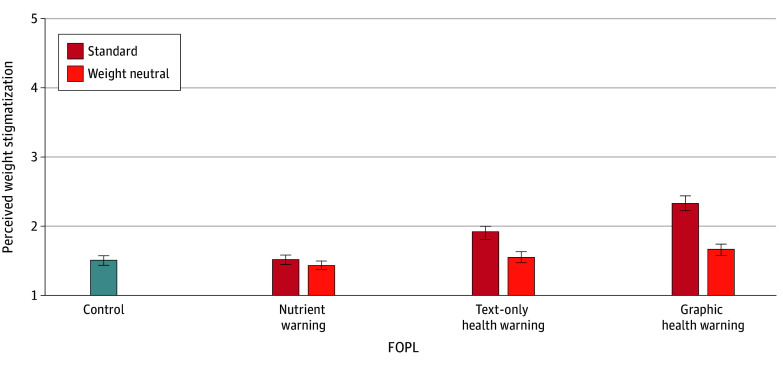
Estimated Mean Perceived Weight Stigmatization by Front-of-Package Label (FOPL) Type Between Participants and Version Within Participants Data are among 2522 participants. Response scale ranges from 1 (low stigmatization) to 5 (high stigmatization). Mean differential effects are given in eTable 5 in Supplement 2. Error bars denote 95% CIs.

When comparing label types in their standard version, nutrient warnings were not perceived as more stigmatizing than control labels (MDE, 0.003 [95% CI, −0.10 to 0.11]; *P* = .96). Text-only and graphic health warnings were perceived as more stigmatizing than control labels (text only: MDE, 0.41 [95% CI, 0.30-0.51]; *P* < .001; graphic: MDE, 0.81 [95% CI, 0.71-0.92]; *P* < .001) and nutrient warnings (text only: MDE, 0.40 [95% CI, 0.30-0.51]; *P* < .001; graphic: MDE, 0.81 [95% CI, 0.70-0.92]; *P* < .001). Graphic health warnings were perceived as more stigmatizing than text-only health warnings (MDE, 0.41 [95% CI, 0.30-0.52]; *P* < .001) (Figure 3 and eTables 4 and 5 in Supplement 2).

When comparing label types in their weight-neutral version, nutrient and text-only health warnings were not perceived as more stigmatizing than control labels or each other. Graphic health warnings were perceived as more stigmatizing than control labels (MDE, 0.15 [95% CI, 0.05-0.26]; *P* = .004), nutrient warnings (MDE, 0.23 [95% CI, 0.12-0.33]; *P* < .001), and text-only health warnings (MDE, 0.11 [95% CI, 0.003-0.22]; *P* = .04) (Figure 3 and eTables 4 and 5 in Supplement 2).

When comparing versions within each label type, participants perceived the weight-neutral versions as less stigmatizing across all label types. The difference in PWS between weight-neutral and standard labels was largest for graphic health warnings (MDE, −0.66 [95% CI, −0.72 to −0.60]; *P* < .001) followed by text-only health warnings (MDE, −0.36 [95% CI, −0.43 to −0.30]; *P* < .001) and nutrient warnings (MDE, −0.08 [95% CI, −0.14 to −0.02]; *P* = .01) (Figure 3 and eTables 4 and 5 in Supplement 2).

### PME

**Figure 4.**
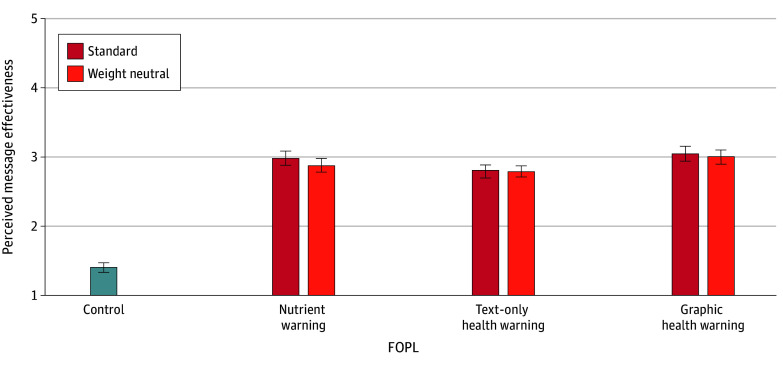
Estimated Mean Perceived Message Effectiveness by Front-of-Package Label (FOPL) Type Between Participants and Version Within Participants Data are among 2522 participants. Response scale ranges from 1 (low effectiveness) to 5 (high effectiveness). Mean differential effects are given in eTable 6 in Supplement 2. Error bars denote 95% CIs.

When comparing label types in their standard version, nutrient warnings (MDE, 1.58 [95% CI, 1.45-1.71]; *P* < .001), text-only health warnings (MDE, 1.41 [95% CI, 1.27-1.54]; *P* < .001), and graphic health warnings (MDE, 1.64 [95% CI, 1.51-1.77]; *P* < .001) were all perceived as more effective than control labels. Nutrient and graphic health warnings did not differ significantly in PME, but both elicited higher PME than text-only health warnings (nutrient warnings: MDE, 0.17 [95% CI, 0.04-0.31]; *P* = .04; graphic health warnings: MDE, 0.24 [95% CI, 0.10-0.37]; *P* = .003) (Figure 4 and eTables 4 and 6 in Supplement 2).

When comparing label types in their weight-neutral version, nutrient (MDE, 1.47 [95% CI, 1.34-1.60]; *P* < .001), text-only (MDE, 1.38 [95% CI, 1.25-1.51]; *P* = .001), and graphic health (MDE, 1.59 [95% CI, 1.46-1.73]; *P* < .001) warnings were all perceived as more effective than control labels. PME did not differ significantly between nutrient and graphic health warnings or between nutrient and text-only health warnings. However, graphic health warnings were perceived as more effective than text-only health warnings (MDE, 0.21 [95% CI, 0.08-0.35]; *P* = .01) (Figure 4 and eTables 4 and 6 in Supplement 2).

When comparing label versions within each label type, participants perceived only some weight-neutral labels as less effective than standard labels and effects were small. Weight-neutral nutrient warnings were perceived as less effective than standard nutrient warnings (MDE, −0.11 [95% CI, −0.16 to −0.06]; *P* < .001), as were weight-neutral compared with standard graphic health warnings (MDE, −0.05 [95% CI, −0.10 to −0.001]; *P* = .048). Weight-neutral and standard text-only health warnings did not differ significantly on PME (Figure 4 and eTables 4 and 6 in Supplement 1).

### Attributional Judgments and Explicit Weight Bias

There were no differences in attributional judgments by label type (eFigure 1 and eTables 4 and 7 in Supplement 2). Participants exposed to nutrient warnings, compared with control labels, exhibited slightly lower levels of weight bias (MDE, −0.08 [95% CI, −0.16 to −0.002]; *P* = .046, while participants exposed to text-only health warnings and graphic health warnings vs control labels did not exhibit different levels of explicit weight bias (eFigure 2 and eTables 4 and 8 in Supplement 2).

### Moderation Analyses

The effects of standard labels on PWS differed based on gender and perceived weight status. Women perceived standard text-only (MDE, 0.53 [95% CI, 0.39-0.67]) and graphic (MDE, 0.95 [95% CI, 0.81-1.09]) health warnings as more stigmatizing than did men or people of another gender (standard text only: MDE, 0.28 [95% CI, 0.14-0.42]; standard graphic: MDE, 0.68 [95% CI, 0.54-0.82]). Individuals who considered themselves slightly or very overweight perceived standard text-only (MDE, 0.46 [95% CI, 0.33-0.60]) and graphic (MDE, 0.95 [95% CI, 0.81-1.09]) health warnings as more stigmatizing than did individuals who considered themselves normal weight or underweight (standard text only: MDE, 0.34 [95% CI, 0.20-0.48]; standard graphic: MDE, 0.69 [95% CI, 0.55-0.82]). By contrast, effects of weight-neutral labels on PWS did not differ based on any characteristics examined (eTable 9 in Supplement 2).

## Discussion

In this randomized clinical trial with US adults, nutrient warnings (regardless of references to calories) were not considered more stigmatizing than control labels but were considered considerably more effective at discouraging sugar-sweetened beverage consumption. When obesity was referenced, text-only and graphic health warnings were perceived as more stigmatizing than control labels and nutrient warnings, with graphic warnings perceived as the most stigmatizing. Alternatively, when obesity was not referenced, perceived stigmatization from both types of health warnings decreased, with no significant difference found for text-only warnings compared with control labels and nutrient warnings. Exposure to any of the FOPLs did not lead to greater attribution of responsibility for weight compared with control labels. Explicit weight bias did not increase with exposure to health warnings and decreased with exposure to nutrient warnings compared with control labels.

Overall, our findings did not reveal a direct trade-off between perceived effectiveness and weight stigmatization. The least stigmatizing labels (ie, nutrient warnings) were perceived as similarly effective as the most stigmatizing (ie, graphic health warnings). Additionally, when health warnings did not reference obesity, perceived stigmatization decreased considerably, but reductions in perceived effectiveness were minimal. Thus, our findings add to a growing evidence base showing that decentering body weight does not necessarily reduce the overall effectiveness of public health messaging. Previous studies showed that weight-neutral messages were more motivating, elicited greater self-efficacy, and even led to healthier food choices than messages focused on weight.^53,67,68,69^

Our finding that nutrient warnings were not perceived as more stigmatizing than control labels diverge from a previous study.^46^ This divergence could be attributable to differences in study stimuli since that study used stop sign–shaped nutrient warnings. Thus, our findings suggest that label design may influence the extent to which nutrient warnings elicit weight stigmatization. Additionally, our finding that graphic warnings did not impact explicit weight bias differs from another previous study.^45^ In both studies, graphic labels referenced the same health effects, and the obesity image depicted the same body part. However, exact images differed, and diabetes was depicted with different body parts. Thus, our findings suggest that small variations in images might affect how much graphic labels elicit weight bias. Alternatively, differences could be due to the measures used in the 2 studies. Our measure focused on salient stereotypes linked to high body weight, which while closely aligned with the negative judgments factor of the scale used in the previous study, did not capture other factors examined in that study (eg, social distance).^45^ In either case, a brief online exposure to FOPLs likely differs from exposure in daily life, which would be prolonged, occur across different settings, and likely lead to social interactions that could influence how labels are interpreted. Therefore, the null effects observed in this study should not be taken as definitive evidence that FOPLs do not impact weight bias. Instead, perceived stigmatization, a more sensitive measure better suited to capture the subtle differences between label types typically observed from brief online exposures, is likely more useful for initially identifying potentially stigmatizing labels.

Together, our findings suggest that policy makers have multiple options for designing FOPLs that effectively influence consumer behavior without stigmatizing body weight. Nutrient warnings seem especially promising for this double aim, a positive development given that nutrient warnings represent the most common type of mandatory FOPL^42^ and are similar to the new FDA-proposed labels.^43^ However, policy makers should assess how specific design elements, such as label shape, might impact weight stigmatization. Additionally, while removing references to calories seems unnecessary for nutrient warnings with nonstigmatizing designs, it could be worthwhile for nutrient warnings with designs that pose a higher risk of stigmatization. Alternatively, should health warnings gain further traction, our findings suggest that excluding general references to body weight and avoiding graphic images depicting obesity may be a promising approach to mitigate stigmatization without largely compromising effectiveness. This approach could also help ensure that health warnings are perceived consistently across population groups regardless of groups’ varying sensitivity to stigmatization due to different experiences with weight stigma.^60,63^

### Strengths and Limitations

This study’s strengths include an experimental design allowing for causal inference, stimuli featuring policy-relevant FOPLs, and a large national sample. Limitations include brief online exposure to FOPLs, which may be insufficient to affect preexisting biases. We also used a convenience sample with higher educational attainment than the US population, limiting generalizability. However, previous evidence suggests that online convenience samples tend to produce experimental results similar in direction to representative samples.^70,71^ Additionally, we examined effects on explicit weight bias, which may differ from those on internalized weight bias (ie, self-stereotyping and self-derogation because of one’s body weight^24^); the latter is worth investigating in the future. We also relied on self-report measures, which may be subject to social desirability bias, although random assignment minimizes the likelihood of confounding by such bias.

## Conclusions

The findings of this randomized clinical trial indicate that FOPLs might discourage unhealthy food consumption but may also contribute to weight stigma. Among different FOPL types, this study found that nutrient warnings performed best at simultaneously maximizing effectiveness and minimizing stigmatization. Labels perceived as more stigmatizing were not consistently perceived as more effective. Policy makers could design effective FOPLs while minimizing stigmatization by, for example, omitting references to obesity from health warnings.

## Notes

Supplement 1.
Trial Protocol

Supplement 2. **eTable 1.** Measures **eTable 2.** Regression Models **eTable 3.** Comparison of Characteristics of the Study With National Estimates **eFigure 1**. Mean Attribution of Responsibility for Body Weight by Label Type **eFigure 2**. Mean Explicit Weight Bias by Label Type **eTable 4.** Output from Regression Models **eTable 5.** Average Differential Effects (ADE) of Label Types and Versions on Perceived Weight Stigmatization (PWS) **eTable 6.** Average Differential Effects (ADE) of Label Types and Versions on Perceived Message Effectiveness (PME) **eTable 7.** Average Differential Effects (ADE) of Label Types on Attribution Of Responsibility for Body Weight **eTable 8.** Average Differential Effects (ADE) of Label Types on Explicit Weight Bias **eTable 9.** Average Differential Effects of Label Types on Perceived Weight Stigmatization Compared to Pooled Control, by Participant Characteristics and Label Version
eReferences 1

eAppendix.

eReferences 2

Supplement 3.
Data Sharing Statement
