# Supplementary material for: Front-of-Package Food Labels and Perceived Weight Stigmatization: A Randomized Clinical Trial
Source: JAMA Netw Open. 2025 Jun 20;8(6):e2516821. doi: 10.1001/jamanetworkopen.2025.16821 (PMC12181792; doi:10.1001/jamanetworkopen.2025.16821)
Supplement: Supplement 1. — Trial Protocol [file jamanetwopen-e2516821-s001.pdf]

Title: Impact of Front-of-Package Labels on Perceived Weight Stigmatization
Protocol Date: November 30th 2023
NCT #: NCT06179043

Introduction

The goal of the analysis described in this document is to use data to be collected through an online experiment to examine the impact of different front-of-package label types and contents on outcomes related to weight stigma and label effectiveness. This document pre-specifies our planned analytic approach prior to data collection.

Study Protocol

Participants will complete an online randomized experiment programmed in Qualtrics. After providing informed consent and completing an initial parent experiment on alcohol messages, participants will be randomly assigned to view 1 of 4 types of front-of-package labels for sugar-sweetened beverages: neutral control labels, nutrient warning labels, text-only health warning labels, or graphic warning labels. In random order, they will view and rate two versions of their assigned label type on perceived weight stigmatization (primary outcome) and perceived message effectiveness (secondary outcome). The two versions will differ only on whether their content references calories or obesity (i.e., the modified version will not reference either). After rating each version of the label, participants will complete measures of weight bias (secondary outcome) and demographic characteristics.

Hypotheses

Primary outcome: We hypothesize that graphic health warnings will be perceived as most stigmatizing, followed respectively by text-only health warnings, nutrient warnings, and control labels (H1). We also hypothesize that, for all label types, the modified version will be perceived as less stigmatizing than the regular version of the same label (H2).

Secondary outcomes: We hypothesize that graphic health warnings will be perceived as most effective, followed respectively by text-only health warnings, nutrient warnings, and control labels (H3). We also hypothesize that graphic health warnings will elicit the most weight bias, followed respectively by text-only health warnings, nutrient warnings, and control labels (H4).

Main Analyses

We will use a two-sided critical alpha of 0.05 to conduct all statistical tests. All confidence intervals presented will use a 95% confidence level. Analyses of the primary and secondary outcomes will include all participants according to the trial arm to which they were randomized. We will use complete case analysis to handle any missing data.

To prepare the data, we will verify that Cronbach's alpha for all scales is sufficient ($\geq$0.7) and, if so, we will average items to create a mean score on each scale for each

participant. If Cronbach's alpha is not sufficient (<0.7), we will drop items as necessary to improve reliability or treat items as separate constructs.

We will first descriptively report unadjusted means for the primary and secondary outcomes for each experimental arm. To test H1 and H2 (model 1) and H3 (model 2), we will fit separate mixed-effects models with random intercepts to account for repeated measures within participants. These models will include indicator variables for label type (i.e., nutrients, text-only, and graphic - the control label type will be omitted as the referent group), an indicator variable for label version (i.e., regular vs. modified), and interaction terms between label type and label version (i.e., nutrients*modified, text-only*modified, and graphic*modified). To test H4 (model 3), we will fit a linear model including only indicators for label type.

If the interaction terms in models 1 and 2 are not significant, after fitting models 1-3, we will use these models to estimate the main average differential effects of label types (i.e., differences in predicted means between label types averaging between the two version of each label). We will obtain the effect of each label type vs. control, as well as of nutrient vs. text-only, text-only vs. graphic, and nutrient vs. graphic label types. If, in turn, the interaction terms in models 1 and 2 are significant, after fitting models 1 and 2, we will estimate the average differential effects of label types and report them separately for each version of the labels (i.e., we will present differences by label type separately for regular labels and for modified labels). We will not adjust the p-value for each label type compared to control, but will adjust for multiple tests for the three additional pairwise comparisons using the Bonferroni-Holm correction.

Exploratory Analyses

We will examine whether participant characteristics (i.e., gender, age, race/ethnicity, and perceived weight status) moderate the effect of label type (compared to control) on the primary outcome. If the main analysis (model 1) reveals that there is no significant interaction by label type and label version, we will fit a series of mixed-effects models with random intercepts (one for each potential moderator) including label type, label version, the moderator, two-way interactions between label type and label version, and two-way interactions between label type and the moderator. If, in turn, the main analysis (model 1) reveals a significant interaction by label type and label version, we will run separate moderation models for regular labels and for modified labels. We will consider moderation to be supported if the interaction terms between label types and the moderator (in either scenario) are statistically significant, and we will probe such significant interactions by calculating the marginal effect of label types on the outcome at different levels of the moderating variables. We will use Wald tests for categorical moderators with more than 2 categories.

Sample Size and Power

This study will occur in a survey that will follow a parent experiment about alcohol messages. The total sample size (~2,500 participants) was calculated based on the primary outcomes of the parent experiment.

Using G*Power3.1 and specifying a between-within subjects ANOVA (which should most closely approximate the proposed mixed linear model), we determined the

minimum effect size we would be able to detect on our primary outcome with this pre-determined sample size. With 80% power, a critical alpha of 0.05, four conditions, and two repeated measures, we would be able to detect an effect of f=0.057 (equivalent to d=0.11) or larger for the between-subjects factor, and f=0.028 (equivalent to d=0.06) or larger for the within-subjects factor. Given that a previous study to analyze the effect of FOPLs on perceived weight stigmatization obtained an effect size of d=0.25 comparing nutrient warnings to control, this study's sample should be sufficiently large to detect an even smaller effect when comparing between nutrient warnings and text-only health warnings, which we expect to be the smallest effect.

Interim Analysis

No interim analyses are planned.
