# Supplementary material for: Front-of-Package Food Labels and Perceived Weight Stigmatization: A Randomized Clinical Trial
Source: JAMA Netw Open. 2025 Jun 20;8(6):e2516821. doi: 10.1001/jamanetworkopen.2025.16821 (PMC12181792; doi:10.1001/jamanetworkopen.2025.16821)
Supplement: Supplement 2. — eTable 1. Measures eTable 2. Regression Models eTable 3. Comparison of Characteristics of the Study With National Estimates eFigure 1. Mean Attribution of Responsibility for Body Weight by Label Type eFigure 2. Mean Explicit Weight Bias by Label Type eTable 4. Output from Regression Models eTable 5. Average Differential Effects (ADE) of Label Types and Versions on Perceived Weight Stigmatization (PWS) eTable 6. Average Differential Effects (ADE) of Label Types and Versions on Perceived Message Effectiveness (PME) eTable 7. Average Differential Effects (ADE) of Label Types on Attribution Of Responsibility for Body Weight eTable 8. Average Differential Effects (ADE) of Label Types on Explicit Weight Bias eTable 9. Average Differential Effects of Label Types on Perceived Weight Stigmatization Compared to Pooled Control, by Participant Characteristics and Label Version eReferences 1 eAppendix. eReferences 2 [file jamanetwopen-e2516821-s002.pdf]

Supplemental Online Content

D'Angelo Campos A, Grummon AH, Ng SW, Puhl R, Golden SD, Hall MG. Front-of-package food labels and perceived weight stigmatization: a randomized clinical trial. *JAMA Netw Open*. 2025;8(6):e2516821. doi:10.1001/jamanetworkopen.2025.16821

eTable 1. Measures
eTable 2. Regression Models
eTable 3. Comparison of Characteristics of the Study With National Estimates
eFigure 1. Mean Attribution of Responsibility for Body Weight by Label Type
eFigure 2. Mean Explicit Weight Bias by Label Type
eTable 4. Output from Regression Models
eTable 5. Average Differential Effects (ADE) of Label Types and Versions on Perceived Weight Stigmatization (PWS)
eTable 6. Average Differential Effects (ADE) of Label Types and Versions on Perceived Message Effectiveness (PME)
eTable 7. Average Differential Effects (ADE) of Label Types on Attribution of Responsibility for Body Weight
eTable 8. Average Differential Effects (ADE) of Label Types on Explicit Weight Bias
eTable 9. Average Differential Effects of Label Types on Perceived Weight Stigmatization Compared to Pooled Control, by Participant Characteristics and Label Version
eReferences 1
eAppendix
eReferences 2

This supplementary material has been provided by the authors to give readers additional information about their work.

© 2025 D'Angelo Campos A et al. *JAMA Network Open*.

eTable 1. Measures

Perceived Weight Stigmatization[2] (α=0.94)
1. This label stigmatizes people with obesity
2. This label promotes negative stereotypes about people with obesity
3. This label portrays people with obesity in a disrespectful manner
Response options: Strongly disagree - Strongly agree
**Perceived Message Effectiveness[3,4]**
How much would this label discourage you from wanting to buy sugary drinks?
Response options: Not at all - A great deal
**Attribution of responsibility for body weight[5] (α=0.85)**
1. People with obesity are responsible for their weight
2. People with obesity are to blame for their weight
Response options: Strongly disagree - Strongly agree
**Explicit Weight Bias[6] (α=0.87)**
1. Lazy - Industrious
2. No will power - Has will power
3. Good self-control - Poor self-control
4. Active - Inactive
5. Self-indulgent - Self-sacrificing
6. Dislikes food - Likes food
7. Undereats - Overeats

© 2025 D'Angelo Campos A et al. *JAMA Network Open*.

eTable 2. Regression models

Outcome	Model
Perceived weight stigmatization (PWS)	$PWS_{ij} = \beta_1 barcode_i + \beta_2 qrcode_i + \beta_3 nutr\_stand_i + \beta_4 nutr\_neutral_i + \beta_5 txt\_stand_i + \beta_6 txt\_neutral_i + \beta_7 graph\_stand_i + \beta_8 graph\_neutral_i + u_{0i} + r_{ij}$<br><br>Stata code:<br>mixed pws i.barcode i.qrcode i.nutr_stand i.nutr_neutral i.txt_stand i.txt_neutral i.graph_stand i.graph_neutral, nocons || responseid:<br>test ((1.nutr_stand + 1.graph_neutral - 1.nutr_neutral - 1.graph_stand) = 0) ((1.txt_stand + 1.graph_neutral - 1.txt_neutral - 1.graph_stand) = 0)
Perceived message effectiveness (PME)	$PME_{ij} = \beta_1 barcode_i + \beta_2 qrcode_i + \beta_3 nutr\_stand_i + \beta_4 nutr\_neutral_i + \beta_5 txt\_stand_i + \beta_6 txt\_neutral_i + \beta_7 graph\_stand_i + \beta_8 graph\_neutral_i + u_{0i} + r_{ij}$<br><br>Stata code:<br>mixed pme i.barcode i.qrcode i.nutr_stand i.nutr_neutral i.txt_stand i.txt_neutral i.graph_stand i.graph_neutral, nocons || responseid:<br>test ((1.nutr_stand + 1.graph_neutral - 1.nutr_neutral - 1.graph_stand) = 0) ((1.txt_stand + 1.graph_neutral - 1.txt_neutral - 1.graph_stand) = 0)
Explicit weight bias (bias)	$bias_i = \beta_0 + \beta_1 control_i + \beta_2 nutr_i + \beta_3 txt_i + \beta_4 graph_i + \varepsilon_i$<br><br>Stata code: reg bias i.nutr i.txt i.graph
Attribution of responsibility for body weight (attr)	$attr = \beta_0 + \beta_1 control_i + \beta_2 nutr_i + \beta_3 txt_i + \beta_4 graph_i + \varepsilon_i$<br><br>Stata code: reg attr i.nutr i.txt i.graph

Note: barcode=barcode control label; qrcode=qrcode control label; nutr=nutrient label; txt=text-only health-effects label; graph=graphic health-effects label; stand=standard version; neutral=neutral version

© 2025 D'Angelo Campos A et al. *JAMA Network Open*.

eTable 3. Comparison of characteristics of the study sample to national estimates

	Study Sample	National Estimate*
	%	*%*
**Gender** (n=2,521)		
Woman	50%	49%
Man	50%	51%
Nonbinary	<1%	N/A
Prefer to self-describe	<1%	N/A
**Race/Ethnicity*** (n=2,521)		
American Indian or Alaska Native, non-Hispanic	<1%	1%
Asian, non-Hispanic	5%	6%
Black or African American, non-Hispanic	13%	9%
Hispanic, Latino, or Spanish, any race	7%	14%
White, non-Hispanic	75%	66%
Other, non-Hispanic	<1%	4%
**Education** (n=2,521)		
Less than high school	1%	10%
High school or GED	10%	27%
Some college or technical school	19%	20%
Associate's degree	11%	8%
Bachelor's degree	41%	21%
Graduate or professional degree	19%	13%
**Household Income** (n=2,519)		
Less than $14,999	6%	7%
15,000 - 24,999	7%	7%
25,000 - $34,999	9%	7%
35,000 - $49,999	14%	10%
50,000 - 74,999	22%	16%
75,000 - $99,999	17%	12%
100,000 - 149,999	16%	17%
150,000 - 199,999	5%	10%
200,000 or more	4%	14%
	Mean (SD)	*Mean (SD)*
**Age** (n=2,522)	44.3 (15.2)	51.00 (19.25)

*National estimates for age, gender/sex, ethnicity, race, and education are survey-weighted estimates among adults (ages 18+) obtained from the 2023 American Community Survey (ACS) 1-year Public Use Microdata Sample (PUMS) (United States Census Bureau, 2024). National estimate of people identifying as non-binary or another gender is listed as "N/A" because the ACS only includes information on identification as male or female. National estimates for income are from the Current Population Survey, 2023, the most recent data currently available.[1]

© 2025 D'Angelo Campos A et al. *JAMA Network Open*.

eFigure 1. Mean attribution of responsibility for body weight by label type (n=2,522)

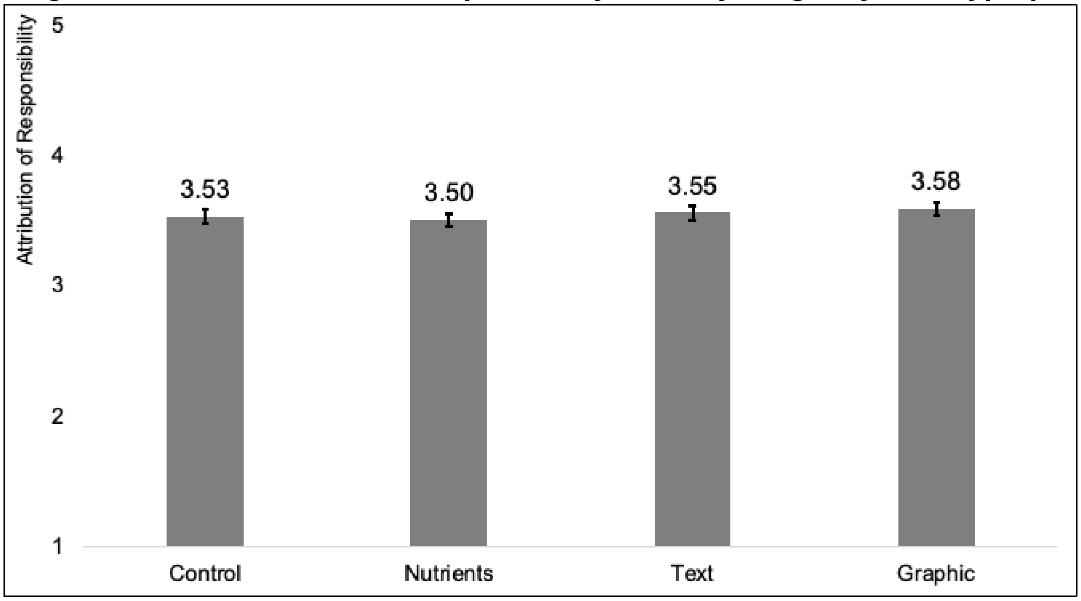

Note: Confidence interval bars denote confidence interval at 95% confidence level

© 2025 D'Angelo Campos A et al. *JAMA Network Open*.

eFigure 2. Mean explicit weight bias by label type (n=2,521)

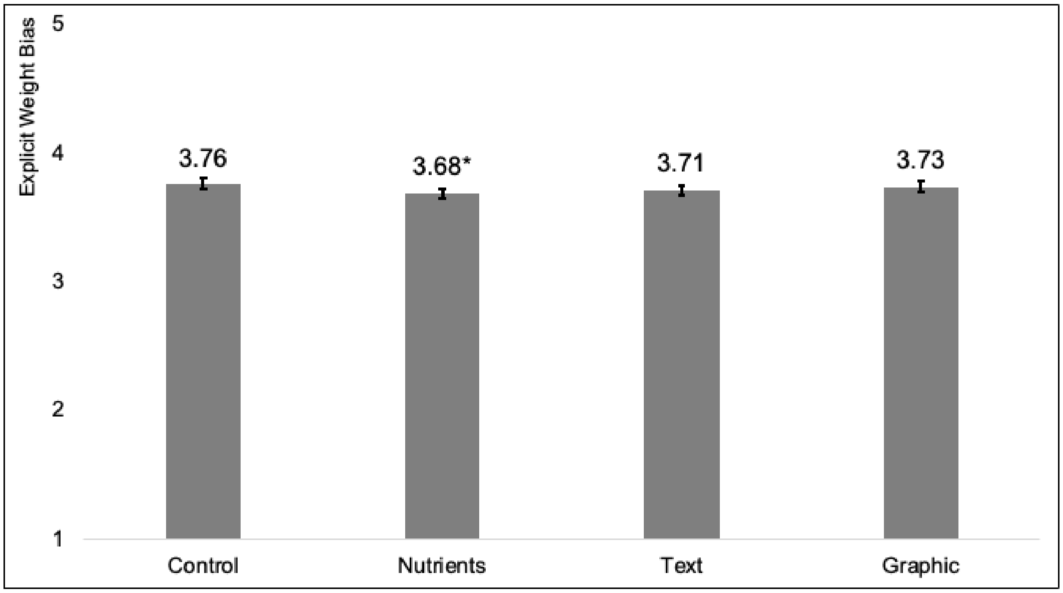

Note: Confidence interval bars denote confidence interval at 95% confidence level
*Significantly different from control at the 95% confidence interval

© 2025 D'Angelo Campos A et al. *JAMA Network Open*.

eTable 4. Output from regression models

	Outcome: Perceived Weight Stigmatization				Outcome: Perceived Message Effectiveness			
	β	*SE*	*95% CI*	*p-value*	*β*	*SE*	*95% CI*	*p-value*
**Barcode**	1.49	0.04	1.42,1.57	<0.01	1.39	0.05	1.29,1.48	<0.01
**QR code**	1.52	0.04	1.45,1.60	<0.01	1.44	0.05	1.35,1.54	<0.01
**Standard nutrient**	1.51	0.04	1.43,1.59	<0.01	2.99	0.05	2.90,3.09	<0.01
**Weight-neutral nutrient**	1.43	0.04	1.36,1.51	<0.01	2.89	0.05	2.79,2.98	<0.01
**Standard text**	1.91	0.04	1.84,1.99	<0.01	2.82	0.05	2.73,2.91	<0.01
**Weight-neutral text**	1.55	0.04	1.47,1.63	<0.01	2.79	0.05	2.70,2.89	<0.01
**Standard graphic**	2.32	0.04	2.25,2.40	<0.01	3.06	0.05	2.96,3.15	<0.01
**Weight-neutral graphic**	1.66	0.04	1.58,1.74	<0.01	3.01	0.05	2.91,3.10	<0.01

	Outcome: Explicit Weight Bias				Outcome: Attribution of Responsibility			
	β	*SE*	*95% CI*	*p-value*	*β*	*SE*	*95% CI*	*p-value*
**Control**	(ref)	(ref)	(ref)	(ref)	(ref)	(ref)	(ref)	(ref)
**Nutrient**	-0.08	0.04	-0.16,-0.002	0.046	-0.03	0.05	-0.13,0.08	0.62
**Text**	-0.05	0.04	-0.13,0.03	0.21	0.03	0.05	-0.08,0.13	0.61
**Graphic**	-0.02	0.04	-0.10,0.05	0.53	0.05	0.05	-0.05,0.16	0.32

© 2025 D’Angelo Campos A et al. *JAMA Network Open*.

eTable 5. Average differential effects (ADE) of label types and versions on perceived weight stigmatization (PWS)

	ADE	SE	p-value
Nutrient *vs.* pooled control			
Standard	0.003	0.05	0.96
Weight-neutral	-0.07	0.05	0.16
Standard	0.41*	0.05	<0.01
Weight-neutral	0.04	0.05	0.43
Graphic health-effects *vs.* pooled control			
Standard	0.81*	0.05	<0.01
Weight-neutral	0.15*	0.05	<0.01
Text-only health-effects *vs.* nutrient			
Standard	0.40*	0.05	<0.01†
Weight-neutral	0.11	0.05	0.07†
Graphic health-effects *vs.* nutrient			
Standard	0.81*	0.05	<0.01†
Weight-neutral	0.23*	0.05	<0.01†
Standard	0.41*	0.06	<0.01†
Weight-neutral	0.11*	0.06	0.04†
Nutrient:<br>weight-neutral *vs.* standard	-0.08*	0.03	0.01
Text-only health-effects:<br>weight-neutral *vs.* standard	-0.36*	0.03	<0.01
Graphic health-effects:<br>weight-neutral *vs.* standard	-0.66*	0.03	<0.01

*Statistically significant at the 95% confidence level
†Adjusted for multiple comparisons (Holm-Bonferroni)

© 2025 D'Angelo Campos A et al. *JAMA Network Open*.

eTable 6. Average differential effects (ADE) of label types and versions on perceived message effectiveness (PME)

	ADE	SE	p-value
Nutrient *vs.* pooled control			
Standard	1.58*	0.07	<0.01
Weight-neutral	1.47*	0.07	<0.01
Standard	1.41*	0.07	<0.01
Weight-neutral	1.38*	0.07	<0.01
Standard	1.64*	0.07	<0.01
Weight-neutral	1.59*	0.07	<0.01
Text-only health-effects *vs.* nutrient			
Standard	-0.17*	0.07	0.04†
Weight-neutral	-0.09	0.07	0.37†
Graphic health-effects *vs.* nutrient			
Standard	0.06	0.07	0.35†
Weight-neutral	0.12	0.07	0.21†
Standard	0.24*	0.07	<0.01†
Weight-neutral	0.21*	0.07	0.01†
Nutrient: weight-neutral *vs.* standard	-0.11*	0.02	<0.01
Text-only health-effects: weight-neutral *vs.* standard	-0.03	0.02	0.31
Graphic health-effects: weight-neutral *vs.* standard	-0.05*	0.02	0.05

*Statistically significant at the 95% confidence level
†Adjusted for multiple comparisons (Holm-Bonferroni)

© 2025 D’Angelo Campos A et al. *JAMA Network Open*.

eTable 7. Average differential effects (ADE) of label types on attribution of responsibility for body weight

	ADE	SE	p-value
Nutrient *vs.* control	-0.03	0.05	0.62
Text-only health-effects *vs.* control	0.03	0.05	0.61
Graphic health-effects *vs.* control	0.05	0.05	0.32
Text-only health-effects *vs.* nutrient	0.05	0.05	0.62†
Graphic health-effects *vs.* nutrient	0.08	0.05	0.39†
Graphic health-effects *vs.* text-only health-effects	0.03	0.05	0.62†

*Statistically significant at the 95% confidence level
†Adjusted for multiple comparisons (Holm-Bonferroni)

© 2025 D'Angelo Campos A et al. *JAMA Network Open*.

eTable 8. Average differential effects (ADE) of label types on explicit weight bias

	ADE	SE	p-value
Nutrient *vs.* control	-0.08*	0.04	0.046
Text-only health-effects *vs.* control	-0.05	0.04	0.21
Graphic health-effects *vs.* control	-0.02	0.04	0.53
Text-only health-effects *vs.* nutrient	0.03	0.04	0.92†
Graphic health-effects *vs.* nutrient	0.06	0.04	0.50†
Graphic health-effects *vs.* text-only health-effects	0.03	0.04	0.52†

*Statistically significant at the 95% confidence level
†Adjusted for multiple comparisons (Holm-Bonferroni)

© 2025 D'Angelo Campos A et al. *JAMA Network Open*.

eTable 9. Average differential effects of label types on perceived weight stigmatization compared to pooled control, by participant characteristics and label version

Standard Label Version							
	p-value for Wald test	Nutrient		Text-only health-effects		Graphic health-effects	
		ADE	*95% CI*	*ADE*	*95% CI*	*ADE*	*95% CI*
**Gender**							
Man, non-binary, or other	**0.03**	0.01	-0,13,0.15	0.28	0.14,0.42	0.68	0.54,0.82
Woman		-0.003	-0.14,0.14	0.53	0.39, 0.67	0.95	0.81,1.09
**Race/Ethnicity**							
Any other race/ethnicity	0.81	0.02	-0.15,0.20	0.36	0.18, 0.54	0.81	0.62,0.999
White		-0.004	-0.13,0.12	0.42	0.30,0.55	0.82	0.69,0.94
**Perceived Weight Status**							
Right weight or underweight	**0.045**	0.02	-0.12,0.16	0.34	0.20,0.48	0.69	0.55,0.82
Slightly or very overweight		-0.01	-0.15,0.13	0.46	0.33,0.60	0.95	0.81,1.09
**Age**							
21-43	0.41	0.06	-0.08,0.20	0.46	0.32,0.60	0.80	0.66,0.94
44+		-0.07	-0.21,0.08	0.36	0.22,0.50	0.82	0.68,0.97

Weight-Neutral Label Version							
	p-value for Wald test	Nutrient		Text-only health-effects		Graphic health-effects	
		ADE	*95% CI*	*ADE*	*95% CI*	*ADE*	*95% CI*
**Gender**							
Man, non-binary, or other	0.16	-0.01	-0.13,0.11	0.01	-0.11,0.13	0.17	0.05,0.29
Woman		-0.13	-0.25,-0.01	0.08	-0.04,0.20	0.14	0.02,0.26
**Race/Ethnicity**							
Any other race/ethnicity	0.59	-0.04	-0.20,0.11	0.001	-0.15,0.15	0.10	-0.06,0.26
White		-0.08	-0.19,0.02	0.06	-0.05,0.16	0.17	0.06,0.27
**Perceived Weight Status**							
Right weight or underweight	0.18	-0.05	-0.17,0.07	-0.03	-0.15,0.09	0.12	0.01,0.24
Slightly or very overweight		-0.10	-0.21,0.02	0.11	-0.01,0.22	0.18	0.06,0.30
**Age**							
21-43	0.53	-0.02	-0.13,0.10	0.12	0.001,0.24	0.18	0.06,0.29
44+		-0.14	-0.26,-0.02	-0.04	-0.15,0.08	0.13	0.01,0.25

Note: Moderator variables were dichotomized to simplify the interpretation of results and maximize statistical power, given the limited number of responses in certain response categories.

© 2025 D’Angelo Campos A et al. *JAMA Network Open*.

eReferences 1

1. Gloria Guzman and Melissa Kollar, U.S. Census Bureau, Current Population Reports, P60-282, Income in the United States: 2023, U.S. Government Publishing Office, Washington, DC, September 2024. https://docs.google.com/viewer?url=https%3A%2F%2Fwww2.census.gov%2Flibrary%2Fpublications%2F2024%2Fdemo%2Fp60-282.pdf

2. Puhl R, Luedicke J, Lee Peterson J. Public Reactions to Obesity-Related Health Campaigns: A Randomized Controlled Trial. *Am J Prev Med*. 2013;45(1):36-48. doi:10.1016/j.amepre.2013.02.010

3. Noar SM, Gottfredson N, Vereen RN, et al. Development of the UNC Perceived Message Effectiveness Scale for Youth. *Tob Control*. Published online December 20, 2021. doi:10.1136/tobaccocontrol-2021-056929

4. Grummon AH, Brewer NT. Health Warnings and Beverage Purchase Behavior: Mediators of Impact. *Ann Behav Med*. 2020;54(9):691-702. doi:10.1093/abm/kaaa011

5. Nutter S, Alberga AS, MacInnis C, Ellard JH, Russell-Mayhew S. Framing obesity a disease: Indirect effects of affect and controllability beliefs on weight bias. *Int J Obes*. 2018;42(10):1804-1811. doi:10.1038/s41366-018-0110-5

6. Bacon JG, Scheltema KE, Robinson BE. Fat phobia scale revisited: the short form. *Int J Obes*. 2001;25(2):252-257. doi:10.1038/sj.ijo.0801537

© 2025 D’Angelo Campos A et al. *JAMA Network Open*.

eAppendix

Original study stimuli available at https://osf.io/zuvtw/files/osfstorage.

Background: Evolutionary psychology posits that social stigmas can emerge in response to three types of determinants: perceived character flaws, perceived physical abnormalities, and out-group membership.[1,2] The strongest known determinant of weight stigma – i.e., the belief that body weight is under personal control and responsibility, also known as attribution of responsibility for body weight[3–13] – is closely related to perceived character flaws attributed to people with higher body weight, such as laziness and lack of self-control.[14,15] Some studies also suggest that a high body weight, which is commonly perceived as a physical abnormality, may trigger individuals' pathogen disgust regardless of the fact that body weight is largely unrelated to infections by pathogens, thus playing a role in weight stigma.[16–22] Lastly, some studies show that exposure to negative social consensuses about a high body weight in one's social groups, which can lead people with high body weight to be perceived as out-group members, can also influence individuals' weight biases.[23–25] This secondary data analysis sought to examine whether attribution of responsibility for body weight, pathogen disgust, and social consensus mediated FOPLs' effects on explicit weight bias.

Measures: All measures used in this study were collected after label exposure (i.e., between-subjects only). We assessed attribution of personal responsibility for body weight with two items adapted from the Blame Attribution scale[26] – i.e., "People with obesity are responsible for their weight" and "People with obesity are to blame for their weight." We assessed pathogen disgust with the single item alluding to personal hygiene from the Antifat Attitudes Test[27] – i.e., "People with obesity are unclean." We assessed social consensus about people with higher body weight with one item adapted from a measure of belief about ingroup social consensus from a previous study[28] – i.e., "A person with obesity would be treated as equal by people in my social groups." The response scale for all mediator variables ranged from "Strongly disagree" (coded as 1) to "Strongly agree" (coded as 5). Lastly, the survey assessed participants' explicit weight bias using seven items from the Fat Phobia Scale.[29] Items reflected salient stereotypes associated with high body weight: laziness, lack of will power and self-discipline, inactivity, gluttony[30] (eTable 1). Participants viewed pairs of antonyms (i.e., lazy vs. industrious, no will power vs. has will power, good self-control vs. poor self-control, active vs. inactive, self-indulgent vs. self- sacrificing, dislikes food vs. likes food, undereats vs. overeats) and chose the point along a five- point spectrum between each pair that best described their beliefs about people with obesity. Responses were coded such that 1 reflected the lowest level of explicit weight bias and 5 reflected the highest level.

Analysis: We conducted a parallel mediation analysis employing the MacKinnon approach.[31] To examine the indirect effects of FOPL types on explicit weight bias, we first fit three separate linear models regressing each of the mediator variables on FOPL types (i.e., the *a pathways*). Next, we fit a fourth linear model regressing explicit weight bias on the three mediators (i.e., the *b pathways*), controlling for FOPL types (i.e., the *c' pathways*). Lastly, we computed a percentile bootstrapped confidence interval for the indirect effects (i.e., *a*b pathways*) using 5,000

© 2025 D'Angelo Campos A et al. *JAMA Network Open*.

repetitions.[32]

Mediation model pathways

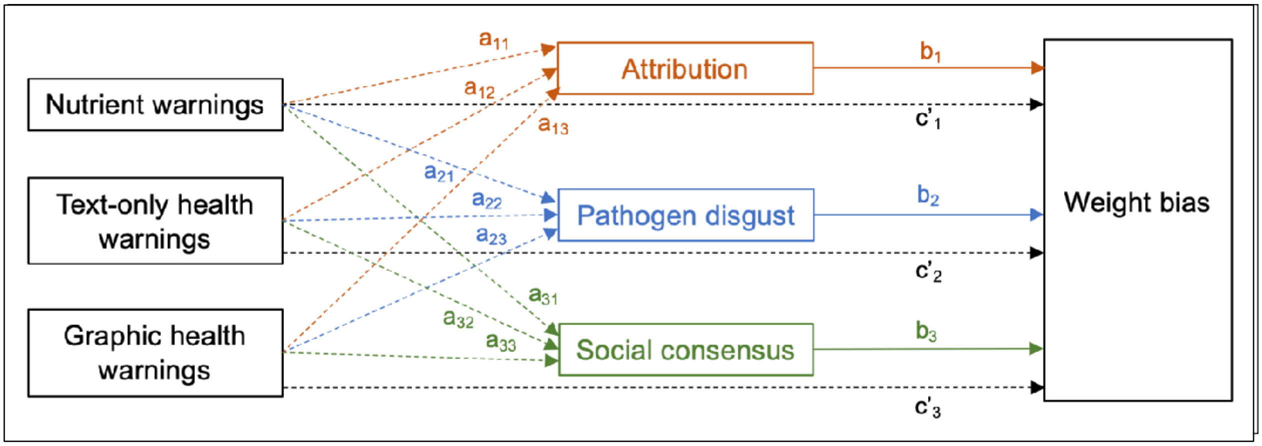

Results: None of the FOPL types examined led to different levels of attribution of responsibility for body weight, pathogen disgust, or social consensus about people with obesity compared to control labels (all p>0.05, Figure 4.3). When examining the associations between mediators and explicit weight bias controlling for FOPL type, we found that higher attribution of responsibility for body weight (p<0.01), higher pathogen disgust (p=0.04), and higher social consensus about people with obesity (p<0.01) were positively associated with higher explicit weight bias (Figure 4.3). Neither attribution of responsibility for body weight, nor pathogen disgust, nor social consensus about people with higher body weight significantly mediated the effects of any of the three types of FOPL on explicit weight bias (Table 4.1).

Mediation pathways of FOPLs' effects on explicit weight bias (n=2,521)

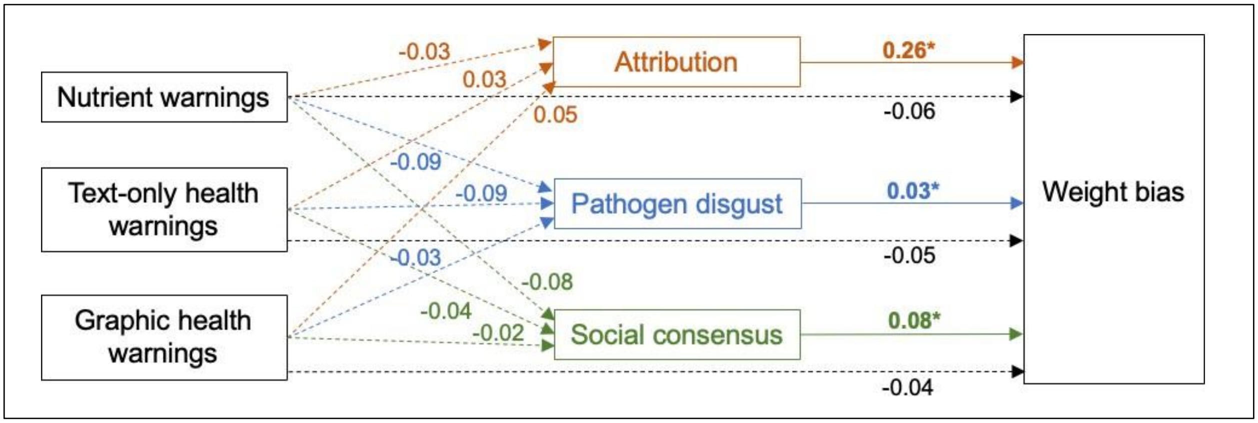

*Statistically significant at the 95% confidence level
Note: Dash lines represent statistically insignificant coefficients while solid lines represent statistically significant coefficients at the 95% confidence level
Note: Coefficients represent change in score on a 5-point scale

© 2025 D'Angelo Campos A et al. *JAMA Network Open*.

Mediation pathways a and b of FOPLs' effects on explicit weight bias (n=2,521)

	a pathway				b pathways			
	β	*SE*	*95% CI*	*p-value*	*β*	*SE*	*95% CI*	*p-value*
**Attribution**					0.26	0.01	0.23,0.29	<0.01
From nutrient warnings	-0.03	0.05	-0.13,0.08	0.62				
From text-only health warnings	0.03	0.05	-0.08,0.13	0.61				
From graphic health warnings	0.05	0.05	-0.05,0.16	0.32				
**Pathogen disgust**					0.03	0.01	<0.01,0.06	0.04
From nutrient warnings	-0.09	0.06	-0.20,0.02	0.11				
From text-only health warnings	-0.09	0.06	-0.20,0.02	0.13				
From graphic health warnings	-0.03	0.06	-0.14,0.08	0.62				
**Social consensus**					0.08	0.01	0.06,0.11	<0.01
From nutrient warnings	-0.08	0.06	-0.21,0.04	0.19				
From text-only health warnings	-0.04	0.06	-0.16,0.08	0.53				
From graphic health warnings	-0.02	0.06	-0.15,0.10	0.72				

Indirect effects of FOPLs through mediators (n=2,521)

	**Indirect effect**	**Bootstrapped 95% CI**
**Nutrients warnings**		
Through attribution (a_{11}*b_1)	-0.007	-0.035,0.022
Through pathogen disgust (a_{21}*b_2)	-0.003	-0.009,0.001
Through social consensus (a_{31}*b_3)	-0.007	-0.018,0.004
**Text-only health warnings**		
Through attribution (a_{21}*b_1)	0.007	-0.021,0.037
Through pathogen disgust (a_{22}*b_2)	-0.003	-0.008,0.001
Through social consensus (a_{23}*b_3)	-0.003	-0.014,0.007
**Graphic health warnings**		
Through attribution (a_{31}*b_1)	0.014	-0.013,0.042
Through pathogen disgust (a_{32}*b_2)	-0.001	-0.005,0.003
Through social consensus (a_{33}*b_3)	-0.002	-0.013,0.009

© 2025 D'Angelo Campos A et al. *JAMA Network Open*.

eReferences 2

1. Kurzban R, Leary MR. Evolutionary origins of stigmatization: The functions of social exclusion. *Psychological Bulletin*. 2001;127(2):187-208. doi:10.1037/0033-2909.127.2.187

2. van Leeuwen F, Hunt DF, Park JH. Is Obesity Stigma Based on Perceptions of Appearance or Character? Theory, Evidence, and Directions for Further Study. *Evol Psychol*. 2015;13(3):1474704915600565. doi:10.1177/1474704915600565

3. Crandall CS. Prejudice against fat people: Ideology and self-interest. *Journal of Personality and Social Psychology*. 1994;66:882-894. doi:10.1037/0022-3514.66.5.882

4. DeJong W. Obesity as a Characterological Stigma: The Issue of Responsibility and Judgments of Task Performance. *Psychol Rep*. 1993;73(3_part_1):963-970. doi:10.1177/00332941930733pt136

5. DeJong W. The Stigma of Obesity: The Consequences of Naive Assumptions Concerning the Causes of Physical Deviance. *Journal of Health and Social Behavior*. 1980;21(1):75-87. doi:https://doi.org/10.2307/2136696

6. Crandall CS, Moriarty D. Physical illness stigma and social rejection. *British Journal of Social Psychology*. 1995;34(1):67-83. doi:10.1111/j.2044-8309.1995.tb01049.x

7. Crandall CS, Martinez R. Culture, Ideology, and Antifat Attitudes. *Pers Soc Psychol Bull*. 1996;22(11):1165-1176. doi:10.1177/01461672962211007

8. Rodin M, Price J, Sanchez F, McElligot S. Derogation, Exclusion, and Unfair Treatment of Persons with Social Flaws: Controllability of Stigma and the Attribution of Prejudice. *Pers Soc Psychol Bull*. 1989;15(3):439-451. doi:10.1177/0146167289153013

9. Wiese HJ, Wilson JF, Jones RA, Neises M. Obesity stigma reduction in medical students. *Int J Obes Relat Metab Disord*. 1992;16(11):859-868.

10. Swift JA, Tischler V, Markham S, et al. Are Anti-Stigma Films a Useful Strategy for Reducing Weight Bias Among Trainee Healthcare Professionals? Results of a Pilot Randomized Control Trial. *OFA*. 2013;6(1):91-102. doi:10.1159/000348714

11. Täuber S, Mulder LB, Flint SW. The Impact of Workplace Health Promotion Programs Emphasizing Individual Responsibility on Weight Stigma and Discrimination. *Frontiers in Psychology*. 2018;9. Accessed February 7, 2023. https://www.frontiersin.org/articles/10.3389/fpsyg.2018.02206

12. Hilbert A. Weight Stigma Reduction and Genetic Determinism. *PLOS ONE*. 2016;11(9):e0162993. doi:10.1371/journal.pone.0162993

13. Prunty A, Clark MK, Hahn A, Edmonds S, O'Shea A. Enacted weight stigma and weight self stigma prevalence among 3821 adults. *Obesity Research & Clinical Practice*. 2020;14(5):421-**427. doi:10.1016/j.orcp.2020.09.003**

© 2025 D'Angelo Campos A et al. *JAMA Network Open*.

14. Crandall CS. Ideology and lay theories of stigma: The justification of stigmatization. In: *The Social Psychology of Stigma*. The Guilford Press; 2000:126-150.

15. Puhl RM, Brownell KD. Psychosocial origins of obesity stigma: toward changing a powerful and pervasive bias. *Obesity Reviews*. 2003;4(4):213-227. doi:10.1046/j.1467-789X.2003.00122.x

16. Miller SL, Maner JK. Overperceiving disease cues: The basic cognition of the behavioral immune system. *Journal of Personality and Social Psychology*. 2012;102(6):1198-1213. doi:10.1037/a0027198

17. Lieberman DL, Tybur JM, Latner JD. Disgust Sensitivity, Obesity Stigma, and Gender: Contamination Psychology Predicts Weight Bias for Women, Not Men. *Obesity*. 2012;20(9):1803-1814. doi:10.1038/oby.2011.247

18. Park JH, Isherwood E. Effects of Concerns About Pathogens on Conservatism and Anti-Fat Prejudice: Are They Mediated by Moral Intuitions? *The Journal of Social Psychology*. 2011;151(4):391-394. doi:10.1080/00224545.2010.481692

19. Park JH, Schaller M, Crandall CS. Pathogen-avoidance mechanisms and the stigmatization of obese people. *Evolution and Human Behavior*. 2007;28(6):410-414. doi:10.1016/j.evolhumbehav.2007.05.008

20. Klaczynski PA. There’s something about obesity: Culture, contagion, rationality, and children’s responses to drinks “created” by obese children. *Journal of Experimental Child Psychology*. 2008;99(1):58-74. doi:10.1016/j.jecp.2007.08.005

21. Park JH, Van Leeuwen F, Chochorelou Y. Disease-Avoidance Processes and Stigmatization: Cues of Substandard Health Arouse Heightened Discomfort With Physical Contact. *The Journal of Social Psychology*. 2013;153(2):212-228. doi:10.1080/00224545.2012.721812

22. Neel R, Neufeld SL, Neuberg SL. Would an Obese Person Whistle Vivaldi? Targets of Prejudice Self-Present to Minimize Appearance of Specific Threats. *Psychol Sci*. 2013;24(5):678-687. doi:10.1177/0956797612458807

23. Puhl RM, Schwartz MB, Brownell KD. Impact of Perceived Consensus on Stereotypes About Obese People: A New Approach for Reducing Bias. *Health Psychology*. 2005;24(5):517-525. doi:10.1037/0278-6133.24.5.517

24. Harper J, Carels RA. Impact of social pressure on stereotypes about obese people. *Eat Weight Disord*. 2014;19(3):355-361. doi:10.1007/s40519-013-0092-1

25. Meaney T, Rieger E. Integrating cognitive dissonance and social consensus to reduce weight stigma. *Body Image*. 2021;37:117-126. doi:10.1016/j.bodyim.2021.02.003

26. Nutter S, Alberga AS, MacInnis C, Ellard JH, Russell-Mayhew S. Framing obesity a disease: Indirect effects of affect and controllability beliefs on weight bias. *Int J Obes*. 2018;42(10):1804- 1811. doi:10.1038/s41366-018-0110-5

27. Lewis RJ, Cash TF, Bubb-Lewis C. Prejudice Toward Fat People: The Development and Validation of the Antifat Attitudes Test. *Obesity Research*. 1997;5(4):297-307. doi:10.1002/j.1550-8528.1997.tb00555.x

© 2025 D’Angelo Campos A et al. *JAMA Network Open*.

28. Farrow CV, Tarrant M. Weight-based discrimination, body dissatisfaction and emotional eating: The role of perceived social consensus. *Psychology & Health*. 2009;24(9):1021-1034. doi:10.1080/08870440802311348

29. Bacon JG, Scheltema KE, Robinson BE. Fat phobia scale revisited: the short form. *Int J Obes*. 2001;25(2):252-257. doi:10.1038/sj.ijo.0801537

30. Puhl RM, Heuer CA. The Stigma of Obesity: A Review and Update. *Obesity*. 2009;17(5):941- 964. doi:10.1038/oby.2008.636

31. MacKinnon DP, Fairchild AJ, Fritz MS. Mediation Analysis. *Annual Review of Psychology*. 2007;58(Volume 58, 2007):593-614. doi:10.1146/annurev.psych.58.110405.085542

32. Hayes AF. *Introduction to Mediation, Moderation, and Conditional Process Analysis: A Regression-Based Approach*. Guilford Publications; 2022. Accessed September 19, 2023. http://ebookcentral.proquest.com/lib/unc/detail.action?docID=6809031

© 2025 D'Angelo Campos A et al. *JAMA Network Open*.
