# Supplementary material for: Front-of-Package Food Labels and Perceived Weight Stigmatization: A Randomized Clinical Trial
Source: JAMA Netw Open. 2025 Jun 20;8(6):e2516821. doi: 10.1001/jamanetworkopen.2025.16821 (PMC12181792; doi:10.1001/jamanetworkopen.2025.16821)
Supplement: Supplement 3. — Data Sharing Statement [file jamanetwopen-e2516821-s003.pdf]

Data Sharing Statement

D'Angelo Campos. Front-of-Package Food Labels and Perceived Weight Stigmatization. *JAMA Netw Open*. Published June 20, 2025. doi:10.1001/jamanetworkopen.2025.16821

Data
Additional Information: https://clinicaltrials.gov/study/NCT06179043 Identifier: NCT06179043
Data available: Yes
Data types: Deidentified participant data
How to access data: We will upload all data to a public Open Science Framework repository upon publication.
When available: With publication

Supporting Documents
Document types: None

Additional Information
Who can access the data: Anyone requesting the data will be able to download them.
Types of analyses: For any purpose.
Mechanisms of data availability: Data will be publicly available to download.
